# Clinical Characteristics of Children With COVID-19: A Meta-Analysis

**DOI:** 10.3389/fped.2020.00431

**Published:** 2020-07-03

**Authors:** Yudan Ding, Haohao Yan, Wenbin Guo

**Affiliations:** Department of Psychiatry, National Clinical Research Center for Mental Disorders, The Second Xiangya Hospital of Central South University, Changsha, China

**Keywords:** SARS-CoV-2, coronavirus disease 2019, children, epidemic, clinical characteristics

## Abstract

**Background:** With the global spread of novel coronavirus disease 2019 (COVID-19), health care systems are facing formidable challenges. Scientists are conducting studies to explore this new disease, and numerous studies have been shared. However, the number of studies on children with COVID-19 is limited, and no meta-analysis of this group has been performed.

**Methods:** A random-effect meta-analysis was conducted to determine the characteristics of children with COVID-19, including their demographic, epidemiological, clinical, laboratory, imaging features, and outcomes. Four databases and reference lists were screened. Percentages were calculated, and pooled prevalence with 95% confidence intervals (CIs) were reported.

**Results:** Of 195 studies, 33 were selected, and 14 (371 patients) of them were included in the meta-analysis. Then, 19 case reports (25 patients) were summarized separately. Our meta-analysis revealed that 17.4% (95% CI = 9.1–27.3) of children had asymptomatic infection. Fever (51.2%, 95% CI = 40.2–62.2) and cough (37.0%, 95% CI = 25.9–48.8) were the most frequent symptoms. The prevalence of severe or critical illness was almost 0% (95% CI = 0–1.0). The most frequent abnormal laboratory findings, in pediatric patients, were leukopenia/lymphopenia (28.9%, 95% CI = 19.5–39.2) and increased creatine kinase (20.1%, 95% CI = 1.3–49.9). Ground-glass opacity was observed in the CT scan of 53.9% (95% CI = 38.4–68.7) of children diagnosed with pneumonia.

**Conclusions:** Children are at a lower risk of developing COVID-19 and have a milder disease than adults. However, the evidence presented in this study is not satisfactory. Further investigations are urgently needed, and our data will be continuously updated.

## Introduction

Novel coronavirus disease 2019 (COVID-19) is caused by severe acute respiratory syndrome coronavirus 2 (SARS-CoV-2) or 2019-nCoV. On March 11, 2020, the World Health Organization recognized this disease as a pandemic. As of 11 June 2020, 7,343,562 confirmed cases, including 416,430 deaths, were reported in more than 150 countries (https://www.ecdc.europa.eu/en/geographical-distribution-2019-ncov-cases). Global health care systems are facing the arduous challenges of this unknown disease, which has drastically attacked human society. This new pathogen, as well as severe acute respiratory syndrome (SARS) coronavirus and Middle East respiratory distress syndrome (MERS) coronavirus, belongs to β-type coronaviruses but has different genetic characteristics (1). Although some studies have indicated a wild-animal origin, the origin of SARS-CoV-2 is still unclear (2). Its main routes of transmission are respiratory droplets and contact, but this virus may also be spread via the fecal–oral route and vertical transmission. In addition to infected patients, asymptomatic cases are important infection sources.

Studies and case reports about COVID-19 have been published. Unrestricted data sharing and cooperation have helped provide solutions to this pandemic. According to a meta-analysis in early March (3), the most predominant clinical manifestations are fever and cough. Dyspnea and myalgia (or fatigue) are also presented in 45.6 and 29.4% of infected patients, respectively. Hypoalbuminemia, lymphopenia, and elevated inflammatory markers, including C-reactive protein, lactate dehydrogenase (LDH), and erythrocyte sedimentation rate (ESR), are the most prevalent laboratory abnormalities. In terms of imaging findings, chest X-ray results of two-thirds of patients have revealed ground-glass opacity (GGO). At least 20% of patients with COVID-19, especially those with comorbidities, need intensive care.

In the context of newly emerging epidemic diseases, a meta-analysis is important to help make clinical decisions because it provides a statistical summary of critical details such as risk factors and clinical, laboratory, and imaging features. However, most studies have focused on adults, and only a small number of pediatric patients with COVID-19 have been reported. According to the Centers for Disease Control and Prevention, as of February 11, 2020, children constituted about 2% of 44,672 confirmed cases in China (4); in the US, children made up 4.5% of 1,663,519 COVID-19 cases, as of June 11, 2020 (https://www.cdc.gov/coronavirus/2019-ncov/cases-updates/cases-in-us.html). Furthermore, in Italy, 1.2% of cases recorded as of March 18, 2020, were children (5). In South Korea, where a highly extensive COVID-19 test was conducted, about 6.4% of all confirmed cases as of April 7, 2020, were children, and no deaths have been recorded (https://www.cdc.go.kr/board/board.es?mid=a30402000000&bid=0030).

Although severe or critical illness and several deaths have been documented in China and other countries, children likely have milder symptoms and a better prognosis. As such, we raise the following questions: What are the demographic, clinical, laboratory, and imaging characteristics of children with COVID-19? Are these findings different from those of adult patients? What is the outcome of infected children? What factors increase the risk of fatal outcomes? We aim to answer these questions through a meta-analysis on children with COVID-19.

## Methods

A prospective protocol was prepared in accordance with the recommendations of the Preferred Reporting Items for Systematic Reviews and Meta-Analysis for study reporting. This protocol has been registered in the International Prospective Register of Systematic Reviews database, and the registration number is CRD42020176942.

### Literature Selection and Data Extraction

A study search was performed on all studies between January 1, 2020, and April 1, 2020, via PubMed, China National Knowledge Infrastructure, Wanfang Database, and Chongqing VIP Database in English or Chinese. No restriction on regions or publication types was imposed. The following terms were used in titles/abstracts: 2019 nCoV/COVID-19/SARS-CoV-2/Novel coronavirus and children/child/infant/teenager/adolescent/pediatric/neonate. Search terms were modified in accordance with the rules of each database. The reference lists of all relevant articles were also screened to broaden our search.

All available peer-reviewed studies or reports (observational studies, cohort studies, case reports, case series, etc.) that reported the risk factors and demographic, clinical, laboratory, and imaging information or outcomes of cases were included. The recruited subjects must have SARS-CoV-2 infection confirmed through real-time reverse transcriptase polymerase chain reaction (RT-PCR). Correspondences, letters, and reviews that reported original data were also included. When several studies described duplicated samples, the most complete study was included. The findings of case reports alone were summarized instead of including them in the meta-analysis. The critical appraisal tool developed by Downes et al. (6) was used to assess the quality of cross-sectional studies. The Quality Appraisal of Case Series Studies Checklist of the Institute of Health Economics (7) was also utilized. The two scales consisted of 20 items, and a score of 0–20 was allocated to each study.

Two authors (Ding and Yan) worked together to select and assess studies, and they discussed any disagreement with the senior author (Guo). They independently extracted the following information: first author; country; type of study; number of cases; demographic (age and sex), epidemiological, clinical (fever, cough, vomiting, etc.), and imaging (computed tomography [CT] of the chest) characteristics; and laboratory results (blood routine and biochemistry).

### Statistical Analysis

A meta-analysis was performed using R version 3.6.3 (meta package). For categorical variables, percentages were calculated, and pooled prevalence with 95% confidence intervals (CIs) were reported to indicate the weighted effect size of each study. For continuous data, mean ± standard deviation (*SD*) was calculated.

The statistical heterogeneity in this meta-analysis was assessed using *I*^2^ statistic, Cochran's Q, and tau-squared test with a random-effect model, and significance was set at *p* < 0.10. Generally, *I*^2^ of no more than 25% means low heterogeneity among studies, and *I*^2^ of more than 75% indicates high heterogeneity (8). Begg's test and funnel plots were performed to inspect publication bias, and the number of the included studies should not be <10.

## Results

### Characteristics of Eligible Studies

**Figure 1:**
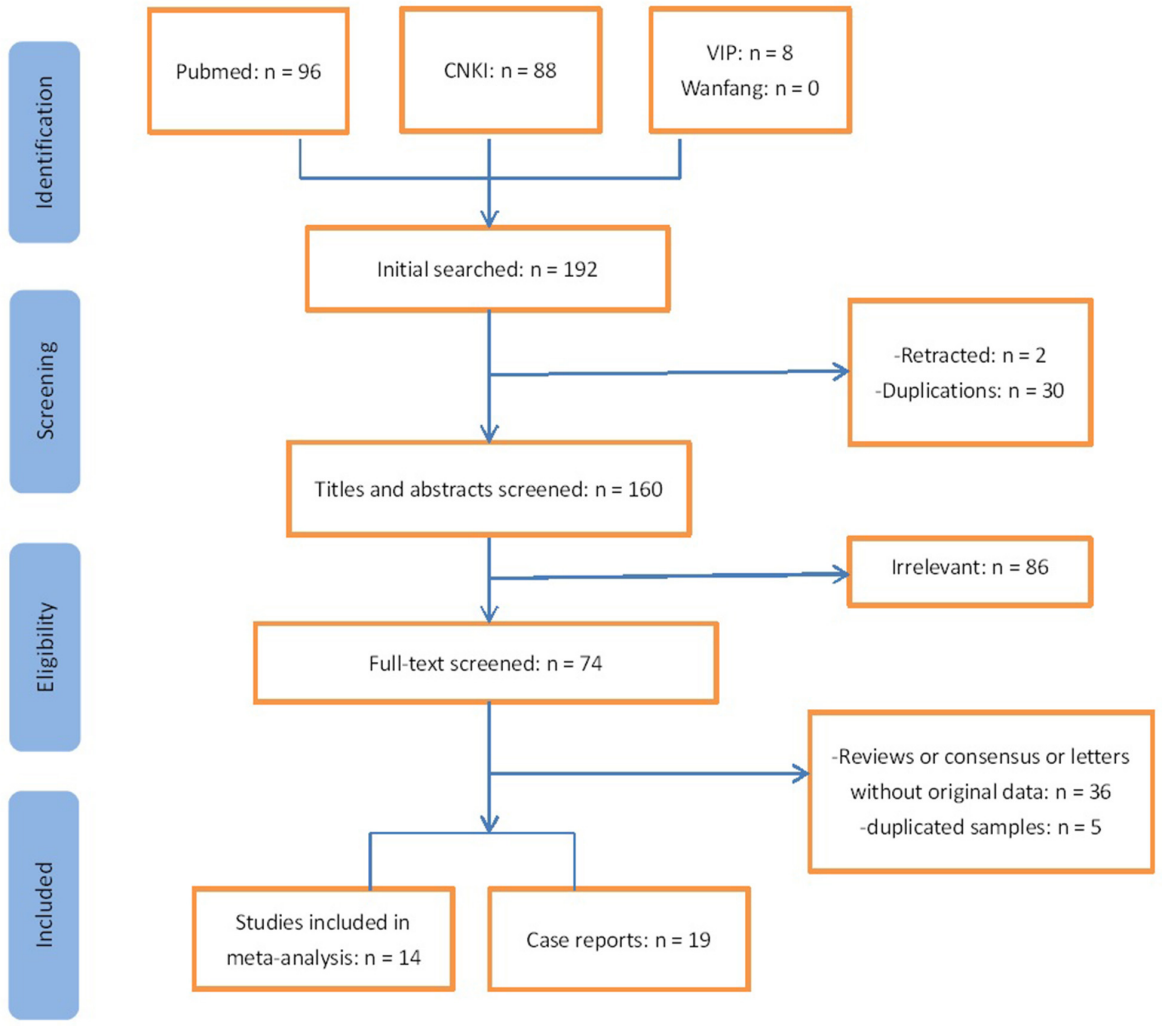
Flow chart of study selection.

In Figure 1, 33 studies, including 396 cases, met the inclusion criteria. Of these studies, 19 were case reports (Table S1), and 14 were included in the meta-analysis (Tables 1–3). A total of 121 articles were excluded from full-text screening, including 33 duplicated studies from different databases, two retracted studies, and 86 irrelevant studies. Then 36 reviews, letters, or consensus without original data were also excluded. Among the remaining studies, we scrutinized the hospitals/institutions and the admission time of recruited patients and ruled out five studies with duplicated samples and incomplete information. All of the studies for the meta-analysis were from China. Half of them were cross-sectional studies and, others were case series. Most of the 19 case reports were also from China and four from Vietnam, Singapore, South Korea, and Thailand, respectively. Lu et al. (9) and Li et al. (10) might have used some of the same data sources. The mean quality score of 14 studies for the meta-analysis was 14 ± 2.4 (ranging from 19 to 11). The journal name and doi of 33 studies and funnel plots are shown in the Supplementary Material.

**Table 1:** Summary of the demographic and epidemiological information of included studies for meta-analysis.

**Study**	**Study type**	**N**	**Male (%)**	**Mean age (y)**	**Age range**	**Quality score**	**Exposure-Family cluster (%)**	**Sever/critical cases/ intensive care (%)**	**Underlying disease (%)**	**Other pathogens^b^ (%)**
Qiu et al.	Cross-sectional	36	23 (64%)	8.3	1y−16y	19	32 (88.9%)	0	NA	NA
Wang et al.	Cross-sectional	31	15 (48%)	7.1	6m−17y	18	28 (90.3%)	0	1 (3.22%)	NA
Li W. et al.	Case series	5	4 (80%)	3	10m−6y	13	4 (80%)	0	NA	NA
Lu et al.	Cross-sectional	171	104 (60.8%)	6.7	1d−15y	17	154 (90%)	3 (1.75%)	10 (10.9%)^a^	7 (7.69%)^a^
Li X. et al.	Cross-sectional	30	18 (60%)	6	0–14y	13	16 (53.3%)	1 (3.33%)	NA	12 (40%)
Zheng et al.	Cross-sectional	25	14 (56%)	3	3m−14y	13	16 (64%)	2 (8%)	2 (8%)	6 (24%)
Feng et al.	Case series	15	5 (33%)	7	4y−14y	12	NA	0	NA	NA
Cai et al.	Case series	10	4 (40%)	6.2	3m−10.9y	15	7 (70%)	0	NA	0
Liu H. et al.	Case series	4	2 (50%)	3.8	2m−9y	13	NA	0	NA	1 (25%)
Zhang et al.	Cross-sectional	10	3 (30%)	4.9	11m−14y	16	10 (100%)	0	NA	NA
Wei et al.	Case series	9	2 (22%)	0.5	1m26d−11m	13	9 (100%)	0	0	NA
Yang et al.	Cross-sectional	11	8 (73%)	8.1	1y2m−15y7m	14	NA	0	NA	0
Liu M. et al.	Case series	5	4 (80%)	6	7m−13y	11	NA	0	NA	NA
Zhong et al.	Case series	9	4 (44%)	6.5	3m−12y	13	9 (100%)	0	0	0

a *The study of Lu et al. did not provide information of patients' underlying disease and other pathogenic infections, so we combined another two studies (with 91 and 115 subjects) which reported duplicated samples but complementary data*.

b *Lu et al. tested respiratory syncytial virus, mycoplasma pneumonia, adenovirus, and influenza virus; Li X et al. tested mycoplasma pneumonia, Legionella pneumophila, EB virus and adenovirus; Zheng et al. tested mycoplasma pneumonia, influenza virus type B and enterobacter aerogenes; Cai et al. tested influenza virus type A and B; Liu H et al. tested mycoplasma pneumonia, influenza virus type A and B, chlamydia pneumonia, coxsackie virus B, human parainfluenza virus, respiratory syncytial virus, and adenovirus; Yang et al. tested mycoplasma pneumonia, influenza virus type A and B, chlamydia pneumonia, Legionella pneumophila, respiratory syncytial virus, and adenovirus; Zhong et al. tested influenza virus type A and B*.

*d, day; m, month; NA, not available; y, year*.

**Table 2:** Summary of the clinical and imaging features of the included studies for meta-analysis.

**Study**	**Asymptomatic infection (%)**	**Pneumonia (%)**	**Fever (%)**	**Cough (%)**	**Dyspnea/tachypnea (%)**	**Pharyngeal congestion/pharyngeal erythema/sore throat (%)**	**Nasal congestion/rhinorrhea (%)**	**Vomiting/diarrhea/abdominal pain (%)**	**Headache/fatigue (%)**	**Imaging-GGO (%)**
Qiu et al.	10 (27.8%)	19 (53%)	13 (36.1%)	7 (19.4%)	1 (2.78%)	3 (8.3%)	0	2 (5.56%)	3 (8.33%)	19 (53%)
Wang et al.	4 (12.9)	14 (45%)	19 (61.3%)	8 (25.8%)	0	5 (16.1%)	2 (6.45)	5 (16.1%)	6 (19.4%)	9 (29%)
Li W. et al.	2 (40%)	0	1 (20%)	1 (20%)^a^	0	1 (20%)	1 (20%)	0	0	3 (60%)
Lu et al.	27 (15.8%)	111 (64.9%)	71 (41.5%)	83 (48.5%)	49 (28.6%)	79 (46.2%)	22 (12.9%)	26 (15.2%)	13 (7.6%)	56 (32.7%)
Li X. et al.	0	30 (100%)	25 (83.3%)	20 (66.7%)	0	1 (3.33%)	2 (6.67%)	4 (13.3%)	2 (6.67%)	28 (93.3%)
Zheng et al.	0	17 (68%)	13 (52%)	11 (44%)	2 (8%)	0	2 (8%)	5 (20%)	0	NA
Feng et al.	NA	12 (80%)	5 (33.3%)	1 (6.67%)	0	0	1 (6.67%)	0	0	7 (46.7%)
Cai et al.	2 (20%)	NA	8 (80%)	6 (60%)	0	4 (40%)	5 (50%)	0	0	4 (40%)
Liu H. et al.	0	3 (75%)	3 (75%)	3 (75%)	0	0	0	0	1 (25%)	1 (25%)
Zhang et al.	0	7 (70%)	4 (40%)	1 (10%)	0	0	1 (10%)	0	0	7 (70%)
Wei et al.	NA	NA	4 (57.1%)^b^	2 (28.6%)^b^	0	0	1 (11.1%)	0	0	NA
Yang et al.	NA	8 (72.7%)	7 (63.6%)	5 (45.4%)	0	2 (18.2%)	6 (54.5%)	2 (18.2%)	0	NA
Liu M. et al.	1 (20%)	NA	2 (40%)	2 (40%)	0	0	0	1 (20%)	0	4 (80%)
Zhong et al.	1 (11.1%)	6 (66.7%)	2 (22.2%)	5 (55.6%)	0	1 (11.1%)	1 (11.1%)	0	0	5 (55.6%)

a *The main symptom of this patient was sputum*.

b *Two of 9 included patients' information were not available*.

*GGO, ground glass opacity*.

**Table 3:** Summary of the laboratory results of the included studies for meta-analysis.

**Study**	**Decreased WBC/lymphocytes (%)**	**Increased WBC/lymphocytes (%)**	**Increased creatine kinase (%)^a^**	**Increased procalcitonin (%)**	**Increased C-reactive protein (%)**	**Increased alanine aminotransferase/aspartate transferase (%)**
Qiu et al.	18 (50%)	0	12 (33.3%)	6 (16.7%)	1 (2.8%)	5 (13.9%)
Wang et al.	4 (12.9%)	7 (22.6%)	4 (12.9%)	1 (2.8%)	3 (9.7%)	6 (19.4%)
Li W. et al.	0	2 (40%)	NA	NA	NA	NA
Lu et al.	51 (29.8%)	0	0	105 (61.4%)	33 (19.3%)	46 (26.9%)
Li X. et al.	13 (43.3%)	0	NA	NA	NA	NA
Zheng et al.	10 (40%)	0	0	NA	0	0
Feng et al.	8 (53.3%)	0	NA	NA	NA	NA
Cai et al.	1 (10%)	4 (40%)	5 (50%)	0	3 (30%)	3 (30%)
Liu H. et al.	1 (25%)	2 (50%)	NA	NA	1 (25%)	NA
Zhang et al.	4 (40%)	5 (50%)	8 (80%)	NA	NA	NA
Wei et al.	NA	NA	NA	NA	NA	NA
Yang et al.	NA	NA	NA	NA	NA	NA
Liu M. et al.	0	0	NA	NA	NA	NA
Zhong et al.	2 (22.2%)	3 (33.3%)	NA	0	0	NA

a *This includes creatine kinase MB*.

*NA, not available*.

### Demographic and Epidemiological Information

The age range of the children in 14 studies was 0–17 years, and their mean age was 5.5 years (*SD*: 2.2; 95% CI = 4.2–6.8). Our results also showed that 58% (95% CI = 47.2–62.2) of the patients were male, and the difference between the number of males and females was not significant (*p* = 0.401). Furthermore, 60.1% (95% CI = 53.7–66.4) of the children were older than 5 years, and significant differences were observed among the number of the three age groups (*p* < 0.001). In addition, 6.1% (95% CI = 2.4–10.9) of all the included children had underlying diseases. In terms of the transmission route, 86.4% (95% CI = 75.5–94.9) of the children with COVID-19 had close contact with family members with COVID-19 (Table 4). Moreover, 10% (95% CI = 0.9–24.2) of the children with COVID-19 tested positive for other pathogens, such as influenza virus types A and B and *Mycoplasma pneumoniae*.

**Table 4:** Results of meta-analysis.

					**Heterogeneity**				**Begg's test^a^**	
**Variables**	***n***	**Prevalence (%)/mean age (years)**	**95% CI**	***N***	***I^**2**^***	**Q**	**Tau^**2**^**	***P***	**t**	***P***
**Epidemiological information**										
Male	14	54.8	47.2–62.2	371	25.1%	17.36	0.0038	0.18	−1.44	0.17
Age (years)	14	5.5	4.2–6.8	371	–	–	–	–	–	–
<1	11	9.0	2.4–1.8	326	64.0%	27.79	0.0192	0.0019	−0.05	0.96
1–5	11	35.3	20.0–54.3	326	82.7%	23.30	1.2676	0.0097	1.52	0.16
>5	10	60.1	53.7–66.4	295	1.4%	9.13	0.0002	0.42	−0.77	0.46
Family cluster	10	86.4	75.5–94.9	336	74.2%	34.92	0.0268	<0.0001	−0.49	0.64
Underlying disease	5	6.1	2.4–10.9	166	0%	3.04	0	0.55	–	–
Other pathogens	7	10.0	0.9–24.2	180	74.8%	23.79	0.0349	0.0006	–	–
**Symptoms and diagnosis**										
Fever	14	51.2	40.2–62.2	369	63.1%	35.28	0.0195	0.0008	0.77	0.46
Cough	14	37.0	25.9–48.8	369	68.3%	41.07	0.0246	<0.0001	−0.86	0.41
Dyspnea/tachypnea	14	1.0	0–7.9	369	79.7%	63.91	0.0446	<0.0001	−3.90	0.002
Pharyngeal congestion/pharyngeal erythema/sore throat	14	8.3	0.4–21.5	367	86.4%	95.89	0.0733	<0.0001	−3.05	0.01
Nasal congestion/rhinorrhea	14	9.9	3.6–18.1	367	62.7%	34.81	0.0193	0.0009	0.73	0.48
Vomiting/diarrhea/abdominal pain	14	7.4	3.4–12.3	369	24.0%	17.11	0.0036	0.1945	−1.95	0.07
Headache/fatigue	14	3.3	1.0–6.4	369	6.6%	13.92	0.0008	0.3796	−1.05	0.31
Asymptomatic infection	10	17.4	9.1–27.3	296	49.9%	17.96	0.0116	0.0356	0.56	0.59
Asymptomatic but had radiologic features of pneumonia	10	19.0	7.4–33.5	296	75.5%	36.68	0.0358	<0.0001	2.22	0.06
Pneumonia	11	66.7	51.1–80.9	347	82.2%	56.22	0.0451	<0.0001	−0.14	0.89
Sever/critical cases/intensive care	14	0	0–1.0	371	0%	4.78	0	0.9797	0.92	0.38
**Imaging**										
GGO	11	53.9	38.4–68.7	326	76.9%	33.03	0.7420	0.0003	2.43	0.04
**Laboratory tests**										
Decreased WBC/lymphocytes	12	28.9	19.5–39.2	351	57.9%	26.14	0.0145	0.0062	−0.56	0.58
Increased WBC/lymphocytes	12	9.1	0.7–22.6	351	85.7%	77.13	0.0631	<0.0001	3.69	0.004
Increased creatine kinase	6	20.1	1.3–49.9	283	94.7%	95.13	0.1310	<0.0001	**–**	**–**
Increased LDH	6	8.3	0–26.1	243	84.8%	32.93	0.0579	<0.0001	**–**	**–**
Increased procalcitonin	5	12.2	0–46.1	257	95.6%	91.02	0.1595	<0.0001	**–**	**–**
Increased C-reactive protein	7	7.4	0.8–17.9	286	72.2%	21.62	0.0221	0.0014	**–**	**–**
Increased alanine aminotransferase/aspartate transferase	5	15.2	4.9–29.1	273	79.5%	19.54	0.0247	0.0006	**–**	**–**

a *Begg's test was conducted when no <10 studies were included*.

*CI, confidence interval; GGO, ground glass opacity; LDH, lactate dehydrogenase; n, the number of included studies; N, the number of included subjects; WBC, white blood cells*.

### Clinical Manifestations and Outcomes

In Table 4, fever (51.2%, 95% CI = 40.2–62.2) and cough (37.0%, 95% CI = 25.9–48.8) were the most frequent symptoms presented by these pediatric patients, whereas 17.4% (95% CI = 9.1–27.3) of the children had asymptomatic infection, that is, they had no symptoms of infection or imaging features of pneumonia. Furthermore, 66.7% (95% CI = 51.1–80.9) of children had pneumonia, and 19% (95% CI = 7.4–33.5) had radiologic features of pneumonia but were asymptomatic. Among 371 cases, only five developed severe or critical illness and required intensive care. Therefore, the result of this meta-analysis was almost 0% (95% CI = 0–1.0). As of April 1, 2020, two child deaths were recorded in China (a 10-month-old child and a 14-year-old boy).

### Laboratory and Imaging Characteristics

The most frequent abnormal laboratory findings in pediatric patients were leukopenia/lymphopenia (28.9%, 95% CI = 19.5–39.2) and increased creatine kinase (20.1%, 95% CI = 1.3–49.9). GGO was observed in CT scans of 53.9% (95% CI = 38.4–68.7) of the children diagnosed with pneumonia (Table 4).

### Case Reports

Nineteen case reports with 25 patients were included. Their mean age was 4.6 years (*SD* = 4.5, range = 0–15 years). Of the 25 patients, 48% were male, and 36% were older than 5 years. Furthermore, 76% of these pediatric patients had a COVID-19 family cluster. No cases had underlying diseases or other pathogenic infections. Common clinical manifestations included fever (60%), nasal congestion/rhinorrhea (28%), cough (24%), and digestive tract symptoms (vomiting/diarrhea/abdominal pain, 24%). In addition, 11 (47.8%) of the 25 patients were diagnosed with pneumonia, four (16%) were asymptomatic but had imaging features of pneumonia, and one (4%) was critically ill. No deaths were reported. Furthermore, five (25%) children had GGO on their CT scan. The most prevalent abnormal laboratory finding was increased creatine kinase (58.3%), followed by increased procalcitonin (55.6%), increased LDH (44.4%), and increased white blood cells/lymphocytes (36.8%; Table S2).

## Discussion

This meta-analysis aimed to provide an evidence-based description of demographic, epidemiological, clinical, laboratory, and imaging features of children with COVID-19 and to assist the management of this group of people (e.g., prioritize limited health resources, minimize loss of life, avoid overtreatment, and shelter vulnerable individuals) in the uncertainty of the ongoing global pandemic.

Consistent with the findings of Dong et al. (11), who summarized the epidemiological characteristics of 2,135 pediatric patients with COVID-19 (728 laboratory-confirmed cases), our results revealed no significant differences between sex, and more than half of the children were older than 5 years. More than 80% of children with confirmed COVID-19 were family cluster cases, and more than 30% of them were asymptomatic. These findings have paramount implications for our social and public health policies. For example, should we re-open childcare centers and schools as soon as possible, given that social distancing, isolation, and the disruption of education all negatively influence children's intelligence development and mental health? Some researchers worry that children might be drivers of household and community transmission, especially when asymptomatic children are looked after by elderly family members who are at a high risk of contracting COVID-19 (12). However, according to recent studies (13), children were less likely to be the index patients and most of them acquired COVID-19 from adults. Secondary infections from infected children were also uncommon. Therefore, children may not be as important in viral transmission as we initially feared. In addition, our findings raise the possibility of underdiagnosis. Li et al. (14) suggested that 86% of all early infections in China were undiagnosed. As such, widespread COVID-19 testing will help us accurately understand the spectrum of this new disease and may lead to the development of solutions for the present morbidity and mortality rates to avoid panic among people (15).

Fever and cough are the two most frequent clinical manifestations presented by children and adults, whereas dyspnea appears to be less frequent in children. Pediatric patients are more likely to show upper respiratory symptoms, such as sore throat, pharyngeal congestion, and rhinorrhea. In contrast to adult patients, only a small number of children with COVID-19 have abnormal laboratory results, and the most predominant findings are leukopenia/lymphopenia and increased creatine kinase instead of increased inflammatory markers (e.g., LDH, ESR, and C-reactive protein) as observed in adults. According to a study focusing only on severe or critically ill children with COVID-19 (16), polypnea and increased inflammatory markers, including C-reactive protein, procalcitonin, and LDH are the most frequently reported symptoms and laboratory features. Lymphopenia is considered a remarkable feature of SARS and MERS because of apoptosis and viral particle-induced cytoplasmic damage (17, 18). However, we have yet to clarify whether COVID-19 is the same. Henry et al. (19) assumed that lymphopenia may be related to the severity of COVID-19, and children (especially very young children) with an immature immune system and different immune responses have a low possibility of having lymphopenia. Similar to adult patients, more than 50% of children have GGO in their imaging results.

Approximately 20% of adult patients need intensive care, and 13.9% have fatal outcomes. Conversely, children with COVID-19 rarely have severe or critical illness, and the number of deaths among them is low. However, children with underlying diseases are still at an increased risk of developing a severe or critical illness. As such, this subgroup of children should be given specific care by their families and health care professionals. As expected, the clinical findings and transmission pattern were consistent regardless of report type. However, the findings of case reports showed a slightly higher infection in females, but the difference was not significant. Compared with the meta-analysis, a proportion of the severe or critical cases was higher in case reports, and increased creatine kinase was the most common laboratory findings. The disparities may be explained by selective bias in case reports.

The reasons why children are less susceptible to COVID-19 and have milder symptoms than those of adults remain elusive. Some hypotheses have been proposed.

First, children and adults have different immune systems. Children's weaker immune responses may prevent pulmonary destruction caused by virus-induced immune responses; similarly, bats possibly have immune tolerance, so they become the natural and healthy reservoir of various viruses (20, 21). Children are vulnerable to having many viral infections, and they can establish innate immune responses when they become infected with respiratory and other RNA viruses, which are associated with innate immune evasion (22). However, whether cross protection works in children who are exposed to other coronaviruses is unclear. In addition, various viruses simultaneously invading the airways and mucosa of the lungs may competitively inhibit SARS-CoV-2 (23). From this point of view, researchers considered that COVID-19 severity may be linked to viral copies (24, 25). However, this hypothesis may not apply to children. In Singapore, a 6-month-old child with a high viral load presented no symptoms except for a single moderate transient fever (26). Some researchers also suggested that children's innate immune responses are more active, and their respiratory tracts are healthier because of their low exposure to cigarette smoke and air pollution (27). Another concern among other researchers is trained immunity. They supposed that a possible reason why children have a mild disease is because of the vaccines that they already received (e.g., Bacille Calmette-Guerin vaccine), training their innate immunity and generate immune memory (28, 29). However, this reason may not explain the disparity of disease severity between children and adults in China, where both groups of people have regular immunizations.

Second, the expression and function of angiotensin converting enzyme 2 (ACE2), a specific viral receptor used by both SARS and SARS-CoV-2, are different. Previous studies suggested that an immature ACE2 receptor limits the binding and infection of these viruses. The ACE2 expression can be induced by angiotensin receptor blockers and ACE inhibitors, which are usually used for adults with hypertension; thus, it may worsen outcomes (25). However, other researchers proposed that ACE2 has protective effects on severe acute lung injury triggered by viral infections (30). Given the mild disease course experienced in most children and the substantial radiation exposure of CT, we should further examine if the use of CT should be routine.

Several limitations of this meta-analysis should be mentioned. First, the number of studies included is small, and most of them are case reports or case series. More short- or long-term clinical studies with comprehensive and rigorous designs are needed. Second, most of the studies included were from China. Studies in other countries are important because of the disparities of ethnicity and lifestyles. For example, the levels of obesity and antenatal smoking in the UK are higher than those in China and Italy, and these factors are correlated with severe viral infection (31). Up to the end of May, several multicenter studies from Italy (32), Spain (33), and the United States (34) have been published. Our preliminary results are consistent with their findings, such as sex ratio, main clinical signs and symptoms, and household or community transmission patterns. However, more asymptomatic children with COVID-19 are reported in our studies than those from Western countries, which may be due to the fact that the rhinopharyngeal swabs were conducted in severely ill patients rather than in asymptomatic individuals in these countries. Comprehensive blood tests and chest radiograph information were not available in their studies. The study from Italy reported that the most common blood investigation was increased C-reactive protein. In addition, more children underwent a chest computed tomography scan in China whereas X-ray or ultrasound was used more frequently in Italy (32). It was intriguing that Kawasaki disease-like symptoms were observed in some children with SARS-CoV-2 infection (positive for nucleic acid or antibodies) in the United States (35) and several European countries (36, 37) due to a disordered immunological response (also called pediatric inflammatory multisystem syndrome temporally associated with SARS-CoV-2, PIMS-TS). SARS-CoV-2 infection triggered systemic inflammatory response, endothelial inflammation and dysfunction through endothelial ACE2, and increased the possibility of Kawasaki disease-like symptoms to develop. Such symptoms were rarely reported in China, thus some researchers speculated that racial and genetic factors may play a crucial role in the symptomatic differences (37). However, a study (38) observed that 24% of 45 children with inflammatory multisystem syndrome, who were confirmed with current or prior SARS-CoV-2 infection, were of Asian origin. Compared with Kawasaki disease and other pediatric inflammatory disorders, according to Whittaker et al. (38), children with PIMS-TS were older, and had more profound lymphopenia and more dominant elevation of inflammatory markers. Compared with our results, children with PIMS-TS included a higher proportion of females, demonstrated more non-respiratory symptoms (especially gastrointestinal symptoms), and reported more increased inflammatory markers. Moreover, increased levels of markers of myocardial injury such as troponin concentrations and N-terminal pro–B-type natriuretic peptide were observed more frequently in children with PIMS-TS, which indicates that a more aggressive treatment plan should be considered such as inotropic support, intravenous immunoglobulin treatment, and corticosteroids treatment. Additionally, COVID-19-associated neurologic and cutaneous manifestations have also been reported worldwide (39–42), indicating a wide clinical manifestation spectrum of this illness. Further studies examining the underlying mechanistic link between SARS-CoV-2 and these manifestations are warranted. Third, many results of these studies, especially laboratory results, are unavailable. Furthermore, laboratory methods and reference ranges vary across different hospitals or research centers, leading to inconsistencies and preventing data integration and comparison.

In conclusion, this meta-analysis reveals the demographic, epidemiological, clinical, laboratory, and imaging characteristics of children with COVID-19. Children are at a lower risk of developing COVID-19 and likely have a milder disease compared with adults. However, the evidence presented in this study is not satisfactory. Further investigations are urgently needed, and our data will be continuously updated.

## Data Availability Statement

All datasets presented in this study are included in the article/Supplementary Material.

## Author Contributions

WG and YD designed the study. YD and HY searched for studies. YD analyzed these included data and wrote the first draft of the manuscript. All authors contributed to and have approved the final manuscript.

## Conflict of Interest

The authors declare that the research was conducted in the absence of any commercial or financial relationships that could be construed as a potential conflict of interest.
