# Supplementary material for: Clinical Characteristics of Children With COVID-19: A Meta-Analysis
Source: Front Pediatr. 2020 Jul 3;8:431. doi: 10.3389/fped.2020.00431 (PMC7350605; doi:10.3389/fped.2020.00431)
Supplement: Supplementary file 1 [file Data_Sheet_1.docx]

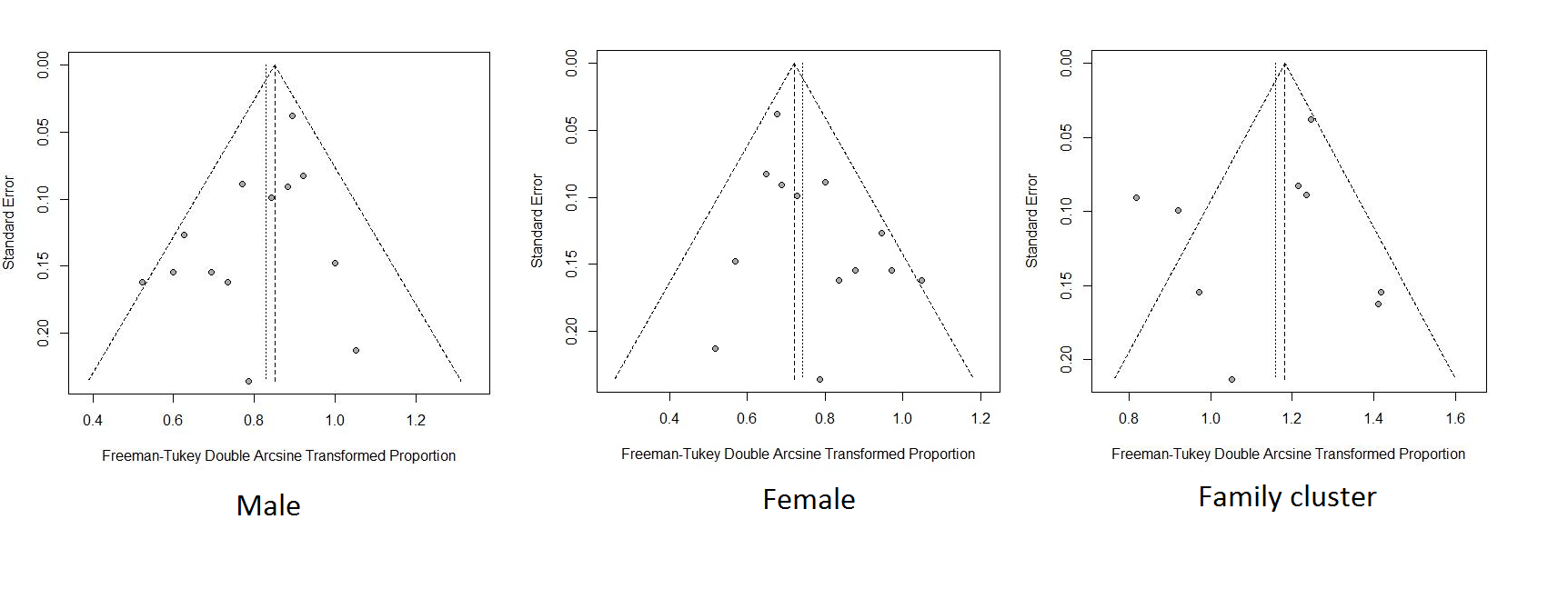


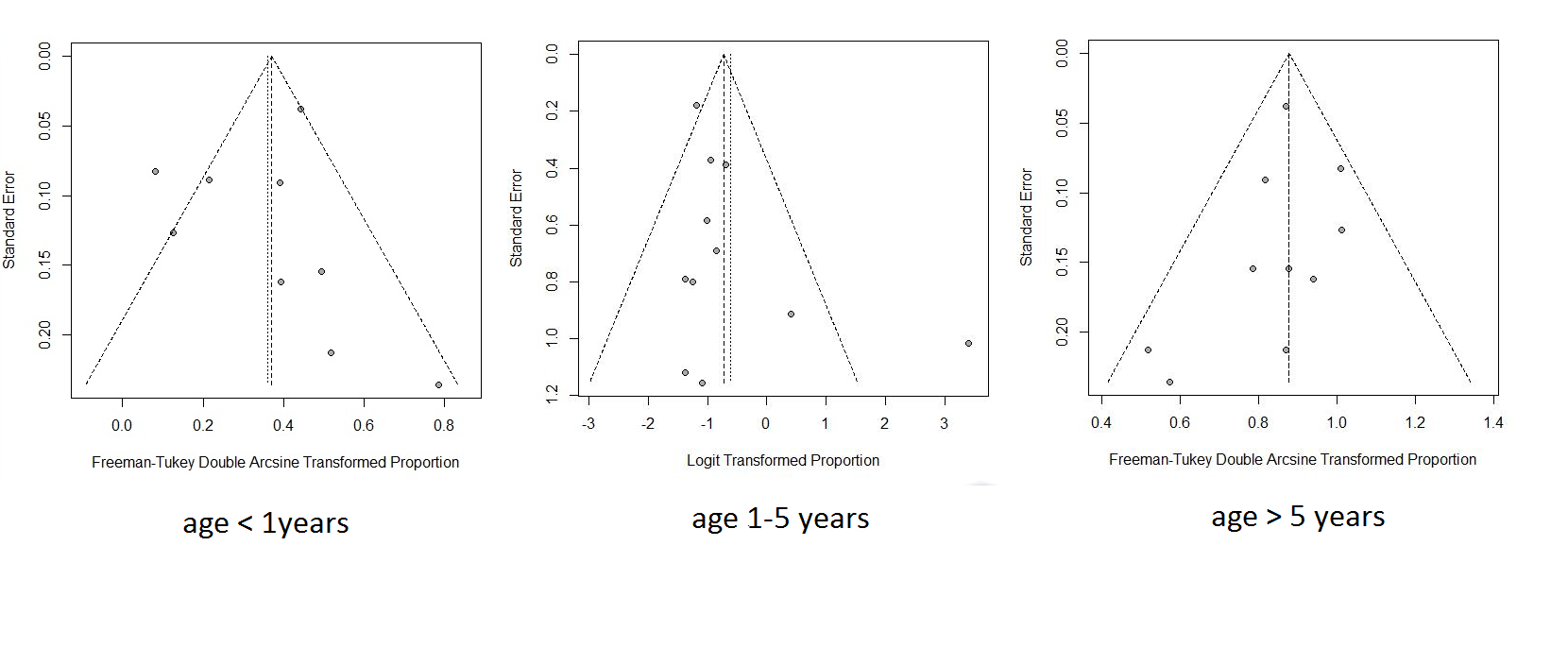


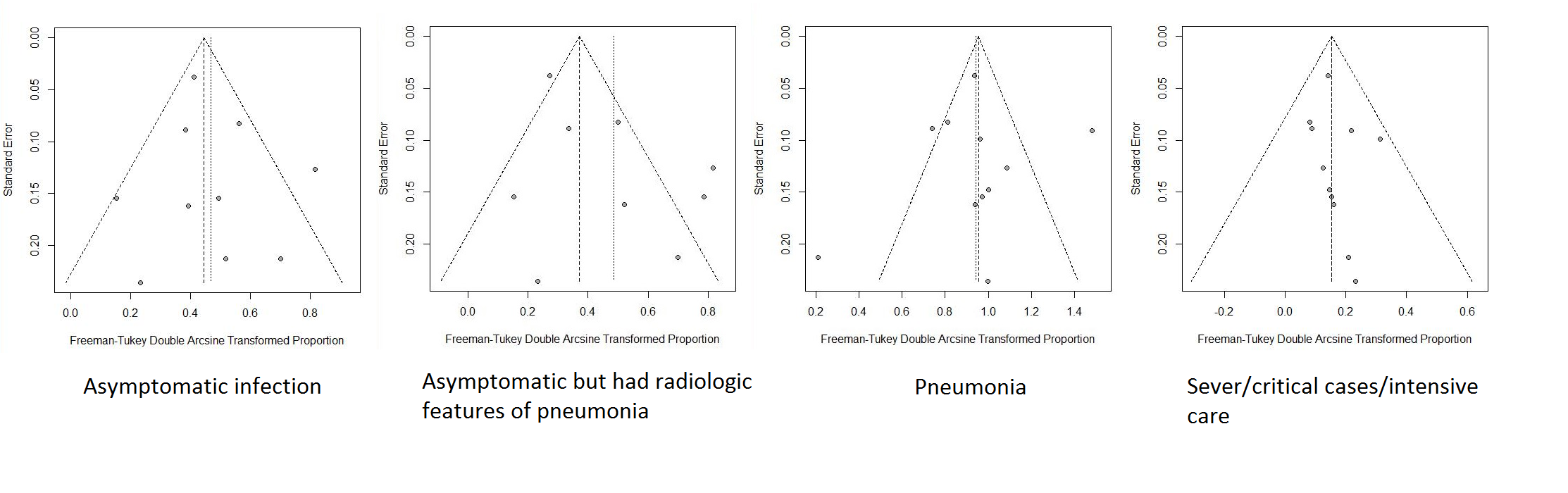


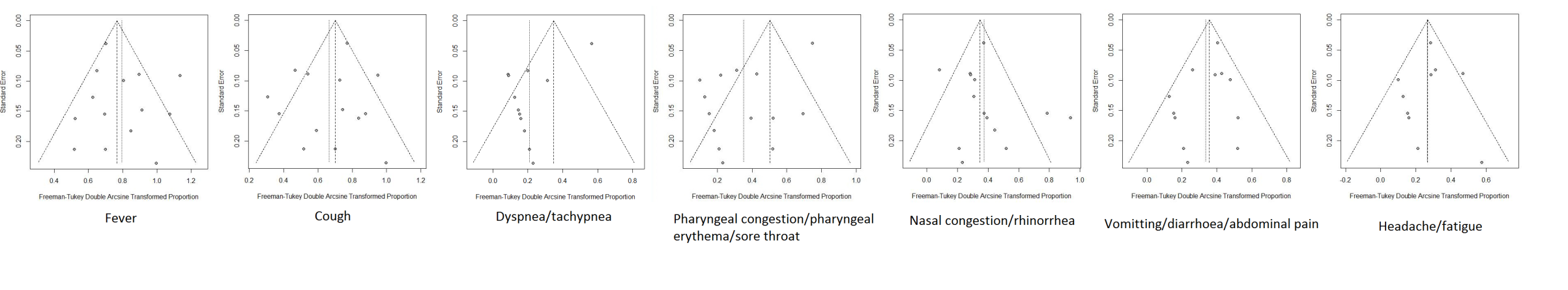


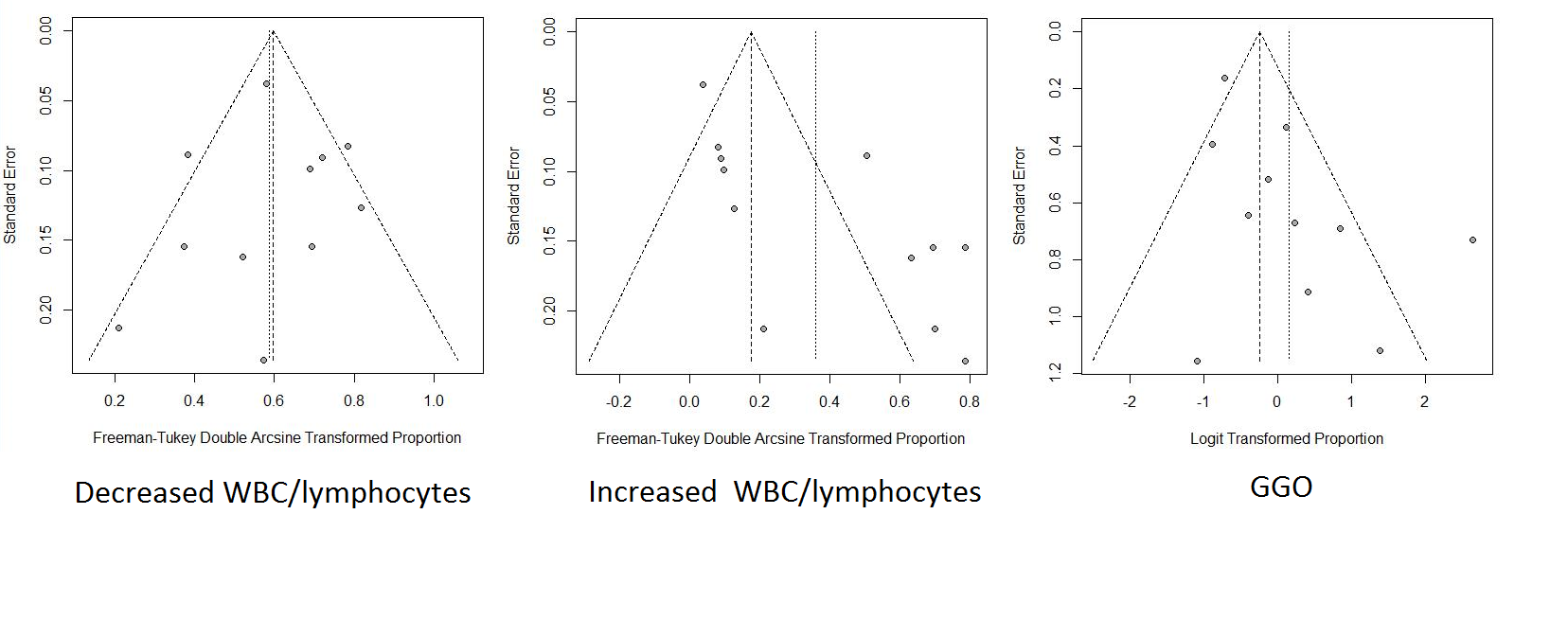


Figure S1. Funnel Plots of results described in Table 4.

Abbreviation: GGO = ground-glass opacity; WBC = white blood cells

Table S1. Basic information of case reports.

| Study | Country | n | Sex | Age |
| --- | --- | --- | --- | --- |
| Ji et al. | China | 2 | 2 male | 15y\9y |
| Li X et al. | China | 1 | female | 3y5m |
| Li Y et al. | China | 2 | 1 male 1 female | Both 4y |
| Xin et al. | China | 3 | 1 male 2 female | 6m\6y\8y |
| An et al. | China | 1 | female | 3y |
| Qian et al. | China | 1 | female | 13m |
| Zhao et al. | China | 1 | male | 13y |
| Zeng et al. | China | 1 | male | 17d |
| Wang et al. | China | 1 | male | 0 (neonate) |
| Le et al. | Vietnam | 1 | female | 3m |
| Kam et al. | Singapore | 1 | male | 6m |
| Yasri et al. | Thailand | 2 | 1 male 1 female | 7y/3y |
| Zhang G et al. | China | 2 | 2 female (twins) | 1y2m |
| Ji et al. | Korea | 1 | female | 10y |
| Cui et al. | China | 1 | female | 55d |
| Chan et al. | China | 1 | male | 10y |
| Chen et al. | China | 1 | male | 1y1m |
| Zhang Y et al. | China | 1 | female | 3m |
| Liu et al. | China | 1 | male | 10y |
| Total: 25 patients, 12 male (48%), age range 0-15y | | | | |

Abbreviation: d = days; m = months; n = the number of patients; y = years.

Table S2. Summary of the findings of case reports.

| Variables | N | Prevalence (%) |
| --- | --- | --- |
| Epidemiological information | | |
| Male | 12 | 48 |
| Age (years) <1 | 7 | 28 |
| 1-5 | 8 | 32 |
| >5 | 9 | 36 |
| Family cluster | 19 | 76 |
| Underlying diseases | 0 | - |
| Other pathogens | 0 | - |
| Symptoms and diagnosis | | |
| Fever | 15 | 60 |
| Cough | 6 | 24 |
| Dyspnea/tachypnea | 1 | 4 |
| Pharyngeal congestion/pharyngeal erythema/sore throat | 1 | 4 |
| Nasal congestion/rhinorrhea | 7 | 28 |
| Vomiting/diarrhea/abdominal pain | 6 | 24 |
| Headache/fatigue | 2 | 8 |
| Asymptomatic infection | 0 | - |
| Asymptomatic but had radiologic features of pneumonia | 4 | 16 |
| Pneumonia | 11 | 47.8 |
| Sever/critical cases/intensive care | 1 | 4 |
| Imaging | | |
| GGO | 5 | 25 |
| Laboratory tests | | |
| Decreased WBC/lymphocytes | 2 | 10.5 |
| Increased WBC/lymphocytes | 7 | 36.8 |
| Increased creatine kinase | 7 | 58.3 |
| Increased LDH | 4 | 44.4 |
| Increased Procalcitonin | 5 | 55.6 |
| Increased C-reactive protein | 3 | 23.1 |
| Increased Alanine aminotransferase/Aspartate transferase | 4 | 26.7 |

Abbreviation: GGO = ground glass opacity; LDH = lactate dehydrogenase; N = the number of included subjects; WBC = white blood cells.

Table S3. Journal and doi of 33 included studies.

| Study | Journal | doi |
| --- | --- | --- |
| Qiu et al. | The Lancet Infectious Diseases | 10.1016/S1473-3099(20)30198-5 |
| Wang et al. | Chinese Journal of Pediatrics | 10.3760/cma.j.cn112140-20200225-00138 |
| Li W et al. | Pediatric Radiology | 10.1007/s00247-020-04656-7 |
| Lu et al. | The New England Journal of Medicine | 10.1056/NEJMc2005073 |
| Li X et al. | Radiologic Practice | 10.13609/j.cnki.1000-0313.2020.03.007 |
| Zheng et al. | Current Medical Science | 10.1007/s11596-020-2172-6 |
| Feng et al. | Chinese Journal of Pediatrics | 10.3760/cma.j.cn112140-20200210-00071 |
| Cai et al. | Clinical Infectious Diseases | 10.1093/cid/ciaa198 |
| Liu H et al. | The journal of Infection | 10.1016/j.jinf.2020.03.007 |
| Zhang et al. | Journal of Shandong University (Health Sciences) | https://kns.cnki.net/KCMS/detail/37.1390.R.20200316.2155.005.html |
| Wei et al. | JAMA | 10.1001/jama.2020.2131 |
| Yang et al. | Journal of Guangzhou University of Traditional Chinese Medicine | http://kns.cnki.net/kcms/detail/44.1425.R.20200318.1641.002.html. |
| Liu M et al. | Journal of Computer Assisted Tomography | 10.1097/RCT.0000000000001023 |
| Zhong et al. | Journal of Central South University (Medicine Science) | 10.11817/j.issn.1672-7347.2020.200206 |
| Ji et al. | World Journal of Pediatrics | 10.1007/s12519-020-00356-2 |
| Li X et al. | Journal of Chongqing Medical University | 10.13406/j.cnki.cyxb.002419 |
| Li Y et al. | Pediatric Pulmonology | 10.1002/ppul.24734 |
| Xin et al. | Journal of Paediatrics and Child Health | 10.1111/jpc.14871 |
| An et al. | Diagnostic and Interventional Radiology | 10.5152/dir.2020.20157 |
| Qian et al. | Clinical Infectious Diseases | 10.1093/cid/ciaa316 |
| Zhao et al. | Zhejiang Medical Journal | http://kns.cnki.net/kcms/detail/33.1109.R.20200218.1956.002.html |
| Zeng et al. | Chinese Journal of Pediatrics | 10.3760/cma.j.issn.0578-1310.2020.0009 |
| Wang et al. | Clinical Infectious Diseases | 10.1093/cid/ciaa225 |
| Le et al. | The Lancet. Child & Adolescent Health | 10.1016/S2352-4642(20)30091-2 |
| Kam et al. | Clinical Infectious Diseases | 10.1093/cid/ciaa201 |
| Yasri et al. | Pediatric Pulmonology | 10.1002/ppul.24737 |
| Zhang G et al. | Chinese Journal of Contemporary Pediatrics | 10.7499/j.issn.1008-8830.2020.03.008 |
| Ji et al. | Journal of Korean Medical Science | 10.3346/jkms.2020.35.e124 |
| Cui et al. | The Journal of Infectious Diseases | 10.1093/infdis/jiaa113 |
| Chan et al. | Lancet | 10.1016/S0140-6736(20)30154-9 |
| Chen et al. | Chinese Journal of Pediatrics | 10.3760/cma.j.issn.0578‐1310.2020.03.003 |
| Zhang Y et al. | Chinese Journal of Pediatrics | 10.3760/cma.j.issn.0578‐1310.2020.03.004 |
| Liu et al. | Science China. Life Sciences | 10.1007/s11427-020-1643-8 |
